# Unmasking portal hypertension: A case of diagnostic distraction by chronic hemoptysis

**DOI:** 10.1016/j.ijscr.2025.111801

**Published:** 2025-08-14

**Authors:** Abdirahman Omer Ali, Mohamoud Hashi Abdi, Hamda Saed Olhayeh, Isra Suleiman Sh. Abdillahi, Amina Ahmed Abdi, Hassan Elmi Moumin

**Affiliations:** a College of Health Sciences, School of Medicine and Surgery, Amoud University, Borama, Somalia; b Borama Regional Hospital, Surgery department, Borama, Somalia; c Borama Regional Hospital, Medical department, Borama, Somalia; d Borama Regional Hospital, Radiology department, Borama, Somalia

**Keywords:** Cavernous transformation of portal vein, Portal hypertension, Esophageal varices, Portal vein thrombosis, Diagnostic challenge, Clinical reasoning

## Abstract

**Introduction and importance:**

The diagnosis of portal hypertension (PHT) secondary to chronic portal vein thrombosis (PVT) relies on recognizing key clinical signs like splenomegaly and cytopenias. Ancillary symptoms, such as chronic hemoptysis, can act as diagnostic distractors, delaying the identification of the underlying pathology. This case report highlights the crucial role of fundamental clinical skills in elucidating a complex diagnosis obscured by a misleading symptom.

**Case presentation:**

A 40-year-old female presented with an eight-year history of hemoptysis, an ancillary symptom that had led to multiple inconclusive pulmonary evaluations. The definitive diagnostic clues were found on our assessment: a palpable spleen on physical examination and severe pancytopenia on routine laboratory tests. These findings established a diagnosis of PHT, which was subsequently confirmed by imaging to be caused by idiopathic chronic PVT, with secondary development of a cavernous transformation of the portal vein (CTPV).

**Clinical discussion:**

The educational value of this case lies in its demonstration of a diagnostic odyssey. The ancillary symptom of hemoptysis obscured the classic signs of PHT for years. The final diagnosis was achieved not by investigating the rare symptom further, but by synthesizing it with the fundamental findings from the physical exam and labs. The hemoptysis, which resolved completely after endoscopic variceal ligation (EVL), was retrospectively explained as a consequence of PHT.

**Conclusion:**

This case serves as a powerful reminder that ancillary symptoms can be misleading. The foundation of an accurate diagnosis often rests upon a thorough physical examination and interpretation of basic laboratory tests, which can reveal the true underlying condition.

## Highlights

• Rare Presentation: A 40-year-old Somali woman presented with chronic hemoptysis, an unusual symptom of CTPV, which typically involves upper gastrointestinal bleeding.
• Diagnosis: She had an eight-year history of recurrent hemoptysis, pallor, and splenomegaly. Lab tests showed pancytopenia. Doppler ultrasound and CT confirmed CTPV with collaterals and splenomegaly; endoscopy revealed esophageal varices.
• Final Diagnosis: Idiopathic CTPV causing portal hypertension, esophageal varices, and hypersplenism, with hemoptysis likely due to variceal bleeding and aspiration.
• Management: Treatment involved blood transfusions, endoscopic variceal ligation (EVL), and non-selective beta-blockers. Anticoagulation was deferred due to bleeding risk, and hemoptysis resolved post-EVL.

## 1 Introduction

The diagnosis of complex systemic conditions, such as portal hypertension (PHT), hinges on astute clinical reasoning and the synthesis of key findings. PHT is frequently caused by chronic portal vein thrombosis (PVT), a blockage of the main portal vein [1]. In response to this chronic obstruction, a network of collateral vessels, known as a cavernous transformation (CTPV), develops to bypass the blockage [2,3]. Therefore, CTPV is not the cause of PHT but rather a key radiological sign of the underlying chronic PVT that is responsible for the elevated portal pressure [[4], [5], [6]].

The classical manifestations of PHT in this non-cirrhotic setting include splenomegaly, hypersplenism (presenting as cytopenias), and potentially life-threatening upper gastrointestinal bleeding (UGIB) from gastroesophageal varices [[7], [8], [9], [10]]. While these signs are well-established, the clinical picture can be significantly clouded by the presence of ancillary symptoms that may misdirect the diagnostic process. Hemoptysis, for example, is an uncommon finding in patients with PHT and almost invariably prompts an investigation focused on primary pulmonary or cardiac etiologies [10]. When it functions as an ancillary, yet dominant, symptom, it can become a powerful diagnostic distractor. This can lead to significant delays in identifying the correct underlying pathology, especially if the more classic signs of PHT are overlooked. This case report describes such a scenario, highlighting how the ultimate diagnosis depended on appreciating the primacy of fundamental clinical findings. This report is written in line with the SCARE 2023 guideline [11].

## 2 Case presentation

A 40-year-old female was referred to the emergency department with an eight-year history of recurrent hemoptysis. This ancillary symptom, consisting of small-volume (<5–10 mL) blood expectoration, had prompted numerous intermittent evaluations at local clinics focused on her respiratory system, all of which were inconclusive. These workups had reportedly included several normal chest X-rays and empirical treatments for bronchitis, without resolution. A comprehensive systemic evaluation had never been performed.

On our presentation, the key diagnostic clues were identified through a basic clinical assessment. Vital signs were stable: blood pressure 120/80 mmHg, heart rate 80 bpm, respiratory rate 16 breaths/min, temperature 37.0 °C, and SpO2 98 % on room air. Marked conjunctival pallor was evident. The cardiopulmonary exam was normal, with clear lung fields bilaterally. However, the abdominal examination revealed a non-tender, firm spleen palpable 4 cm below the left costal margin. There was no ascites, jaundice, or other stigmata of chronic liver disease.

Initial laboratory investigations provided the definitive evidence needed to shift the diagnostic focus. A complete blood count showed severe pancytopenia (WBC 3.7 × 10^9/L, Hgb 7.2 g/dL, Platelets 51 × 10^9/L), consistent with hypersplenism. Therefore, a working diagnosis of significant portal hypertension was confidently made based on this classic pairing of splenomegaly and pancytopenia, independent of the chronic hemoptysis.

**Fig. 1:**
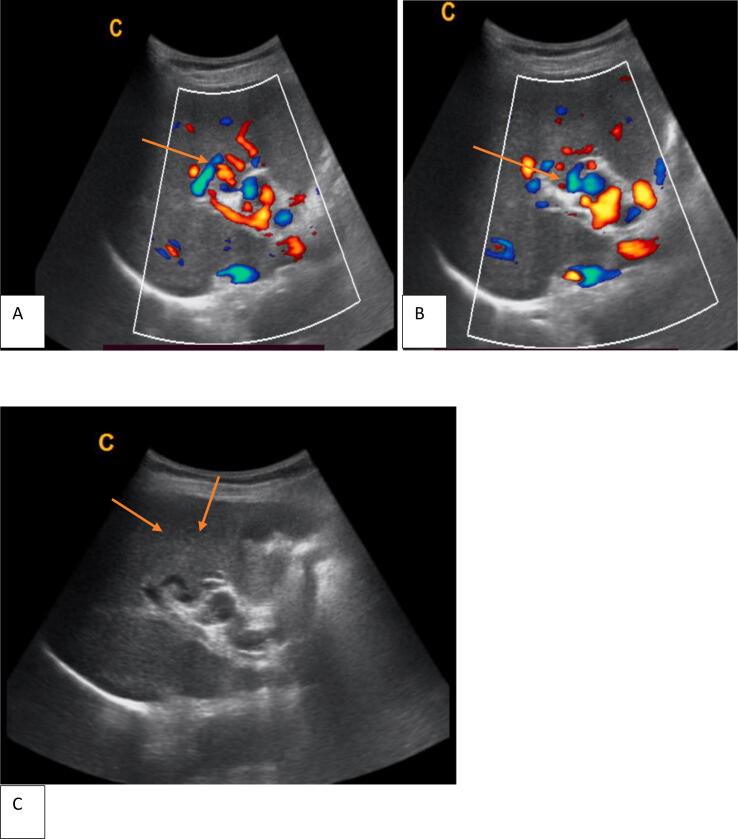
Transverse RUQ gray scale and color Doppler images show. The main portal vein cannot be identified, with the portal vein bed containing multiple clusters of serpiginously enhancing vessels extending into the liver (Orange arrows in panels A, B, AND C).

**Fig. 2:**
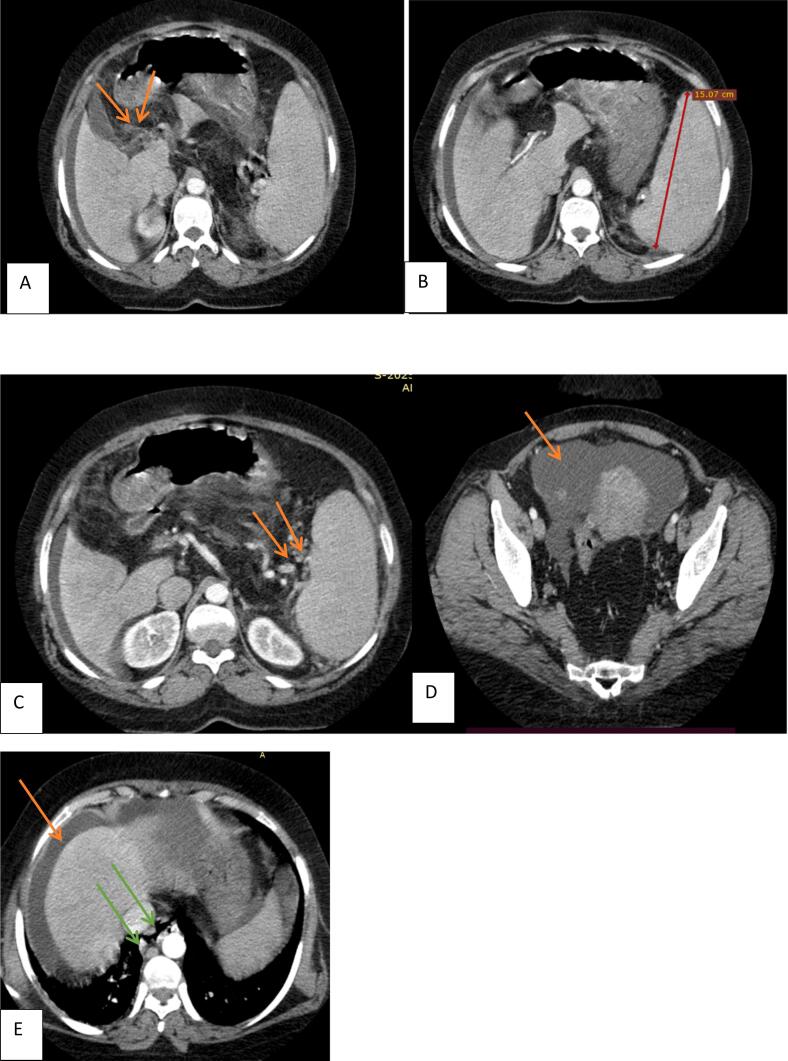
A,B,C and D. Multiple poorly Enhancing lesions with beaded appearance are seen at the porta hepatis with no obvious main portal vein (orange arrows in A) suggesting collaterals which have replaced the main portal vein. There are also multiple torturous, enlarged, smooth enhancing tubular structure in peri-splenic and at the esophagus (orange arrows in C and green arrows in E respectively). There is hypertrophy of liver segment IV and Caudate lobe and atrophy of left lobes Moderate ascitic fluid seen suggesting early cirrhosis with portal hypertension.

Imaging was then employed for confirmation and etiological assessment. Abdominal Doppler ultrasound and contrast-enhanced CT confirmed the underlying pathology: a chronically thrombosed main portal vein, with a resulting network of collateral vessels consistent with CTPV (Fig. 1, Fig. 2). Marked splenomegaly and extensive porto-systemic collaterals were also noted. Upper endoscopy identified Grade II-III esophageal varices with high-risk stigmata (red wale signs).

The final diagnosis was established as Idiopathic Chronic Portal Vein Thrombosis, which had resulted in non-cirrhotic Portal Hypertension and subsequent Cavernous Transformation.

## 3 Therapeutic intervention and management

Initial management focused on hemodynamic stabilization and correction of anemia, with the patient receiving two units of packed red blood cells. Following endoscopic confirmation, definitive therapy was undertaken via endoscopic variceal ligation (EVL) of the identified esophageal varices. A critical management consideration arose regarding anticoagulation. After multidisciplinary discussion, the decision was made to prioritize variceal bleeding prophylaxis by initiating a non-selective beta-blocker (Propranolol) and to defer systemic anticoagulation due to the high bleeding risk from severe thrombocytopenia and high-risk varices.

### 3.1 Outcome and follow-up

Following EVL, the patient's episodes of hemoptysis resolved completely and did not recur. Her hemoglobin level stabilized with iron therapy. She was discharged on Propranolol with a plan for surveillance endoscopy. At 6-month follow-up, she remained free of hemoptysis, although pancytopenia persisted due to hypersplenism.

## 4 Discussion

The most clinically pertinent insight from this case is not the rare association between hemoptysis and PHT, but the demonstration of how an ancillary symptom can obscure a diagnosis that is ultimately revealed by fundamental clinical skills. For eight years, the hemoptysis served as a diagnostic distractor, leading to a prolonged and fruitless search for a pulmonary cause. The diagnostic impasse was broken only when attention was paid to the findings of a thorough physical examination and a basic blood count.

This case powerfully illustrates the concept of diagnostic synthesis. The patient's clinical picture was a puzzle with several pieces: a misleading ancillary symptom (hemoptysis), a crucial physical sign (splenomegaly), and definitive laboratory evidence (pancytopenia). The diagnosis was not possible by focusing on any single piece. Instead, it required the clinician to step back and assemble all the findings into a coherent whole, revealing the underlying syndrome of PHT caused by chronic PVT. The hemoptysis was subsequently explained retrospectively; its complete resolution after EVL provides strong evidence that it was a rare manifestation of variceal bleeding, likely via micro-aspiration. While the initial diagnostic approach for hemoptysis correctly focuses on pulmonary or cardiovascular disease [4,6,13], this case highlights the need to re-evaluate when those pathways are unrevealing and other systemic clues are present.

Another key educational point is the nuanced management of non-cirrhotic PHT secondary to chronic PVT. The decision to prioritize bleeding prophylaxis with EVL and beta-blockers while deferring anticoagulation—despite the thrombotic origin of the disease— highlights the critical need for risk stratification in these patients [5,9,14]. This remains a challenging area requiring individualized clinical judgment based on bleeding risk versus thrombosis risk [10,12].

## 5 Conclusion

This case serves as a powerful reminder that while ancillary symptoms can be distracting, the foundation of an accurate diagnosis often rests upon a thorough physical examination and the careful interpretation of basic laboratory tests. Recognizing classic disease patterns, such as the link between splenomegaly and cytopenias in portal hypertension secondary to portal vein obstruction, is a cornerstone of clinical medicine that enables clinicians to see past confounding factors and arrive at the correct diagnosis.

## CRediT authorship contribution statement

Dr. Abdirahman Omer Ali, Dr. Hassan Elmi Moumin, Isra Suleiman Sh Abdillahi Nour^,^ Dr. Amina Ahmed Abdi and Dr. Hamda Saed Olhayeh individuals contributed to taking history and providing care to the patient throughout his hospital stay. Additionally, Dr. Abdirahman Omer Ali and Dr. Hassan Elmi Moumin contributed to the development of the manuscript. Dr. Mohamoud Hashi Abdi is the radiologist.

## Patient consent

Written informed consent was obtained from the patient for publication of this case report and accompanying images. A copy of the written consent is available for review by the Editor-in-Chief of this journal on request.

## Ethical approval

This case report is exempt from ethical approval.

## Guarantor

Abdirahman Omer Ali, on behalf of all authors, accept full responsibility for the work.

## Ethical considerations

The study protocol, case investigation, and consent form were thoroughly examined by the institutional review board of the College of Health Sciences at Amoud University. They granted approval for the study, along with the Ministry of Health and Borama Hospital in Awdal Region, Somaliland (BRH-215/2024). Prior to participation, written informed consent was obtained from every individual involved.

## Registration of research studies

Not applicable.

## Funding

We had no sponsor nor funding for the writing of the case report.

## Declaration of competing interest

The authors report no declarations of interest.
